# A comparison of three bone graft struts for interbody fusion using a posterior approach for lower lumbar spinal tuberculosis in adults: a midterm follow-up study

**DOI:** 10.1186/s12891-022-05539-8

**Published:** 2022-06-18

**Authors:** Zhenchao Xu, Xiyang Wang, Zhen Zhang, Dingyu Jiang, Runze Jia, Yilu Zhang

**Affiliations:** 1 Department of Spine Surgery and Orthopaedics, the Xiangya Hospital of Central South University, 87# Xiangya Road, Changsha, Hunan 410008 People’s Republic of China; 2 Hunan Engineering Laboratory of Advanced Artificial Osteo-Materials, 87# Xiangya Road, Changsha, Hunan 410008 People’s Republic of China

**Keywords:** Posterior approach, Autogenous bone graft, Allogeneic bone graft Titanium mesh cage, Lower lumbar spinal tuberculosis

## Abstract

**Background:**

This retrospective observational study was conducted to compare midterm outcomes of three bone graft struts for interbody fusion using a posterior approach in adults with lower lumbar spinal tuberculosis.

**Methods:**

A total of 126 lower lumbar spinal tuberculosis patients were treated by one-stage posterior debridement, interbody fusion, and instrumentation. Forty-one patients (group A) were treated with autogenous bone graft for interbody fusion, 45 patients (group B) were treated with allogeneic bone grafting, and the remaining 40 (group C) patients were treated with titanium mesh cage. In addition, clinical and radiographic data were gathered and analyzed.

**Results:**

At the final follow-up, all patients were completely cured. The operation period and intraoperative blood loss for groups B and C were significantly less than in group A (*P* = 0.000). Post-operation, neurological performance and quality of life were remarkably improved at the final follow-up. The preoperative lordosis angles of three groups were significantly improved, as evidenced by the values immediately after the operation or those at the final follow-up. The correction loss of the group C was lower than those of groups A and B (*P* = 0.000). All the patients obtained bone graft fusion, the fusion period of group B was longer than that of the other two groups (*P* = 0.000). No significant differences among the three groups in adjacent segment degeneration rates were found at the last visit (*P* = 0.922).

**Conclusions:**

This midterm follow-up study established that one-stage posterior debridement, interbody fusion, and instrumentation, combined with medical therapy, can effectively treat lower lumbar spinal tuberculosis. In addition, the intervertebral titanium mesh cage bone graft can provide better outcomes with regard to maintaining lordosis and preventing collapse.

**Supplementary Information:**

The online version contains supplementary material available at 10.1186/s12891-022-05539-8.

## Background

Spinal tuberculosis (TB), the most common extrapulmonary TB, accounts for about 50% of bone and joint TB [1]. This severe spinal disease often causes spinal instability, kyphosis, spinal nerve dysfunction, or paraplegia [2]. Tuberculosis of the lower lumbar spine (L3–L5) is not uncommon in clinical practice. It has the characteristics of high refractory, disability, and recurrence rate, which seriously influences the quality of patients' life. Anti-TB drugs are used across the entire treatment period; a reasonable surgical treatment improves the cure rate [3].

The optimal surgical approach for lower lumbar spinal TB in adults is still controversial [4, 5]. As per the conventional surgical method, the anterior approach or combined posterior and anterior procedures are preferred. Nevertheless, increased complications due to complex anatomical structure, and trauma were often linked to surgical procedures.

Based on literature reviews, posterior debridement, interbody fusion, and instrumentation can improve clinical effects [6, 7]. Often, TB of the spine annihilates the vertebral body, and late stages erode the intervertebral spaces and diminish the stability. Therefore, reconstruction of the anterior and middle columns of the spine is advantageous in curing the lower lumbar spinal TB and preventing recurrence. Although various types of bone graft struts have been suggested for the interbody fusion of TB spondylitis and satisfactory results were obtained [8, 9], treatment of lower lumbar spinal TB with allogeneic, autogenous, and/or titanium mesh cage bone grafts has not been reported. Therefore, this single-centre retrospective cohort study compares the efficacy of the three bone graft struts for interbody fusion for treating lower lumbar spinal TB using a posterior approach.

## Methods

### Patient data

The inclusion criteria for this study were as follows: (1) Lesions limited to one or two adjacent segments without extensive TB abscess were included (in case of multiple segments being involved, only one or two vertebral bodies needed to be addressed surgically); (2) Severe or progressive neurological impairment; (3) Spinal instability or deformity, progressive aggravation of the trend; (4) Persistent low back pain with no benefits with medications.

Exclusion criteria included individuals who had: (1) undergone lower lumbar surgery; (2) a history of congenital scoliosis, deformity or ankyloses; (3) multilevel large psoas abscess or gravity abscess; and (4) patients with surgical contraindications were evaluated preoperatively. Patients were followed up for at least five years with complete data.

A total of 126 patients with lower lumbar spinal TB were treated with one-stage posterior debridement, interbody fusion, and instrumentation from January 2004 to December 2014. Of them, 75 were male, and 51 were female patients. The average age of the patients at surgery was 47.4 ± 13.1 years. The number of lesion segments treated in these individuals was one in 24 and two in 102. Three types of interbody bone grafts were performed in this study: 41 patients underwent autogenous and intervertebral bone grafts to get the anterior and middle columns reconstructed (Group A); 45 patients were treated with allogeneic bone grafts for reconstruction (Group B), and the rest of the 40 patients were treated with titanium mesh cage bone grafting (group C). Unfortunately, it was difficult to randomly select a surgical treatment method in clinical practice. Therefore, in our study, all patients in Group C were collected more recently, the patients in group B were collected earlier, and patients in group A were collected earliest period.

The individuals' clinical symptoms included lower-back pain, weakness, low fever, and varying degrees of lower limb dysfunction. The Erythrocyte sedimentation rate (ESR) and C-reactive protein (CRP) values were raised to varying degrees. Preoperative diagnosis was conducted based on serological examination and imaging outcomes, comprising spinal radiographic films, computed tomography (CT), and magnetic resonance imaging (MRI), which exhibited vertebral bone destruction, narrowing or disappearance of intervertebral spaces, and cold abscesses. The neurologic examination was conducted as per the Japanese Orthopedic Association (JOA) score. The Oswestry Disability Index (ODI) was utilized to assess the quality of life. Back pain and radicular pain of the lower extremity were estimated with the help of the Visual Analogue Scale (VAS). The University of California at Los Angeles (UCLA) grading scale [10] was applied to evaluate the adjacent segment degeneration (ASD) on the radiograph.

No significant differences among the three groups in the variables such as gender, age, diseased vertebrae number, preoperative ESR, CRP, JOA, ODI, VAS, and preoperative lordosis angles (Table 1) were found.

**Table 1:** Preoperative data of patients

	Group A	Group B	Group C	Statistical value	P_A–B_/P_A–C_/P_B–C_
Gender (Male/Female)	24/17	27/18	24/16	χ^2^ = 0.025, *P* = 0.988	-/-/-
Age (years)	47.6 ± 13.3	45.6 ± 13.6	49.4 ± 12.3	F = 0.905, *P* = 0.407	-/-/-
Diseased vertebrae number	1.9 ± 0.5	1.8 ± 0.5	1.9 ± 0.5	F = 0.486, *P* = 0.616	-/-/-
During of symptoms (months)	3.0 ± 1.2	3.3 ± 1.4	3.6 ± 1.5	F = 1.378, *P* = 0.256	-/-/-
ESR (mm/h)	68.4 ± 18.3	71.0 ± 16.0	69.9 ± 19.7	F = 0.223, *P* = 0.800	-/-/-
CRP (mg/L)	42.6 ± 10.5	46.8 ± 16.2	45.6 ± 15.8	F = 0.968, *P* = 0.383	-/-/-
JOA	18.8 ± 3.5	18.2 ± 3.8	18.3 ± 3.7	F = 0.279, *P* = 0.757	-/-/-
ODI	42.7 ± 5.9	43.6 ± 6.1	42.4 ± 7.2	F = 0.387, *P* = 0.680	-/-/-
VAS	6.9 ± 1.0	7.1 ± 1.1	6.8 ± 1.2	F = 0.620, *P* = 0.540	-/-/-
Lordosis angle (°)	16.1 ± 4.5	15.9 ± 4.5	16.3 ± 5.2	F = 0.079, *P* = 0.924	-/-/-

### Preoperative management

All patients received anti-TB drugs 2 to 4 weeks prior to the surgery, including isoniazid (5 mg/kg/day, < 300 mg/day), rifampicin (10 mg/kg/ day, < 1200 mg/day), and pyrazinamide (30 mg/kg/day, < 2000 mg/day), and ethambutol (15 mg/kg/day, < 2500 mg/day). They were strictly advised to bed rest, strengthen their nutritional intake, and get anaemia and hypoproteinemia corrected simultaneously. Only when the symptoms of TB subside or disappear (the ESR and body temperature returned to normal or decreased significantly, and anemia and hypoalbuminemia were corrected) surgery may be conducted. During the anti-TB period, surgery may be performed in the presence of acute paralysis or progressive aggravation of neurological impairment, even if the ESR value does not decline.

### Surgical method

The surgery was conducted with the patient lying in a prone position under general anesthesia.

A posterior midline incision was made considering the diseased vertebral body to be the center, in group C, exposing bilateral lamina, facet joints, and transverse processes. Pedicle screws were fixed in one or two vertebrae adjacent to the affected vertebrae, and short pedicle screws were also installed in the affected vertebrae if the pedicle screw channel was not destroyed by infection. A hemilaminectomy or laminectomy was conducted on the highly damaged side of the lesion segment. Then, the diseased vertebral bodies were exposed by removing the superior and inferior articular processes and pedicle. With the help of curettes of different angles, the lesion tissues including the sequestrum, necrotic intervertebral disc, caseous necrosis, and pus were removed, through the transpedicular space, until blood exuded on the bone surface. Thereafter the silicone tube was carefully placed deep into the lesion along the sinus tract, and the pus was absorbed under negative pressure. The procedure was repeated on the other side of the lesion if required. Installation of permanent rods and exerting compression with the help of a cantilever bending maneuver under the vision to correct the deformity and scoliosis were performed. Both the upper and lower bone surfaces of the vertebral body were repaired as bone graft beds. One or two ideally shaped titanium mesh cages filled with autogenous bone particles (from the healthy lamina and spinous process) were used on both sides. Allogeneic bone particles (from strip-shaped allogeneic bone into granular shape with rongeur forceps) in the middle of the basis of the shape and size of the bone graft bed to reconstruct the anterior middle column. Moreover, autogenous and allogeneic granular bones were implanted between bilateral transverse processes, or a suitable allogeneic bone plate was placed between the vertebral lamina. Streptomycin powder (1 g) and isoniazid (0.3 g) were applied in the lesion area, and the incision was closed in layers by placing a drainage tube.

A small incision was made from the posterior superior iliac spine of the patient, and iliac bone blocks of appropriate size were taken, trimmed and implanted into the bone graft bed in group A. The rest of the surgical procedures were the same as those followed in group C.

In group B, allogeneic bone blocks were trimmed to the appropriate size and then implanted into the bone graft bed. Other procedures were the same as those in group C.

Mycobacterium culture and histopathological examinations were carried out on the focus tissues of each patient during the operation.

### Postoperative management

Routine antibiotics were administered and nutritional support was provided post-operation. The drainage tube was removed once the drainage volume collected in 24 h was less than 30 ml. All the patients were continued to be administered with anti-TB drug chemotherapy regimen post-operation mentioned earlier for 12 to 18 months. Routine blood, liver function tests, ESR, and CRP evaluations were conducted to observe the adverse reactions and assess the efficacy of drugs. Following strict bed rest post-operation for four weeks, patients were permitted to walk gradually with the help of an external brace for six months. Early rehabilitation training and physical therapy should be imparted to all patients to prevent thrombus and improve neurological function. Clinical and radiologic examinations were conducted once every three months during the first year post-operation in all patients and once in every six months thereafter.

### Evaluating standard and statistical analysis

The operation period, intraoperative bleeding amount, and fusion period for each group of patients were documented. Bone healing was gauged as per the radiologic criteria of Lee et al. through CT [11]. The following indexes were recorded preoperatively, postoperatively, and during the follow-up: (1) ESR and CRP; (2) neurological status according to JOA; (3) ODI and VAS; (4) lordosis angle; (5) ASD according to UCLA grading scale; and (5) surgery-related complications. Various outcome measures were defined as follows: correction loss = postoperative lordosis angle –final follow up lordosis angle; correction rate = (postoperative lordosis angle – preoperative lordosis angle)/ postoperative lordosis angle.

SPSS 20.0 software was used for performing statistical analysis. The data of the three groups were compared by way of variance analysis first, followed by the LSD⁃*t* test to compare each group when the value of *P* < 0.05. The data were statistically analyzed with the chi-square test. *P* < 0.05 was considered statistically significant.

## Results

### Clinical data

Pathological examinations of all surgical specimens confirmed tuberculous granuloma or caseous necrosis, of which 36 cases were cultured positive for *Mycobacterium tuberculosis*. The follow-up periods for the groups A, B, and C were 75.4 ± 11.8 months, 76.5 ± 11.2 months, and 76.0 ± 11.5 months, respectively. All the patients diagnosed with lower lumbar spinal TB were tested to be clinically cured at the final follow-up.

The operation period and intraoperative blood loss were, respectively, recorded as 189.1 ± 27.2 min and 946.3 ± 185.2 ml in group A, 161.8 ± 24.6 min and 788.9 ± 139.8 ml in group B, 163.3 ± 23.3 min and 777.5 ± 130.6 ml in group C. The results indicated that the values of group A were greater than those of groups B and C (*p* < 0.05). The ESR and CRP values normalized at three months post-surgery.

Patients suffering from preoperative neurological dysfunction exhibited improvement post-surgery in both groups. At the final follow-up, the JOA, ODI, and VAS values were recorded to be 27.1 ± 1.8, 9.9 ± 1.5, and 0.9 ± 0.8 in group A; 27.3 ± 2.0, 10.0 ± 1.7, and 0.9 ± 0.7 in group B; and 27.3 ± 1.9, 10.2 ± 1.7, and 0.9 ± 0.7 in group C. Statistically significant differences were found between preoperative and the final follow-up values of JOA, ODI, and VAS (*p* < 0.05). Nevertheless, VAS values one day postoperatively were higher in group A than those of groups B and C. No significant differences in t JOA, ODI, and VAS were observed among the three groups at the final follow-up.

### Radiographic data

The immediately recorded postoperatively and final follow-up lordosis angles were 29.8 ± 4.3° and 27.0 ± 3.8° in group A, 29.6 ± 4.2° and 26.8 ± 4.6° in group B, 30.7 ± 6.8° and 30.0 ± 6.6° in group C, respectively. The correction loss values were 2.9 ± 1.0°, 3.1 ± 0.8°, and 0.8 ± 0.7 in groups A, B, and C, respectively. The preoperative lordosis angles of the three groups were evaluated to be remarkably rectified compared with immediately recorded postoperatively or at the final follow-up. No significant differences were recorded in the correction rates among the three groups. Nevertheless, the correction loss of group C was observed to be lower than those of groups A and B.

**Fig. 1:**
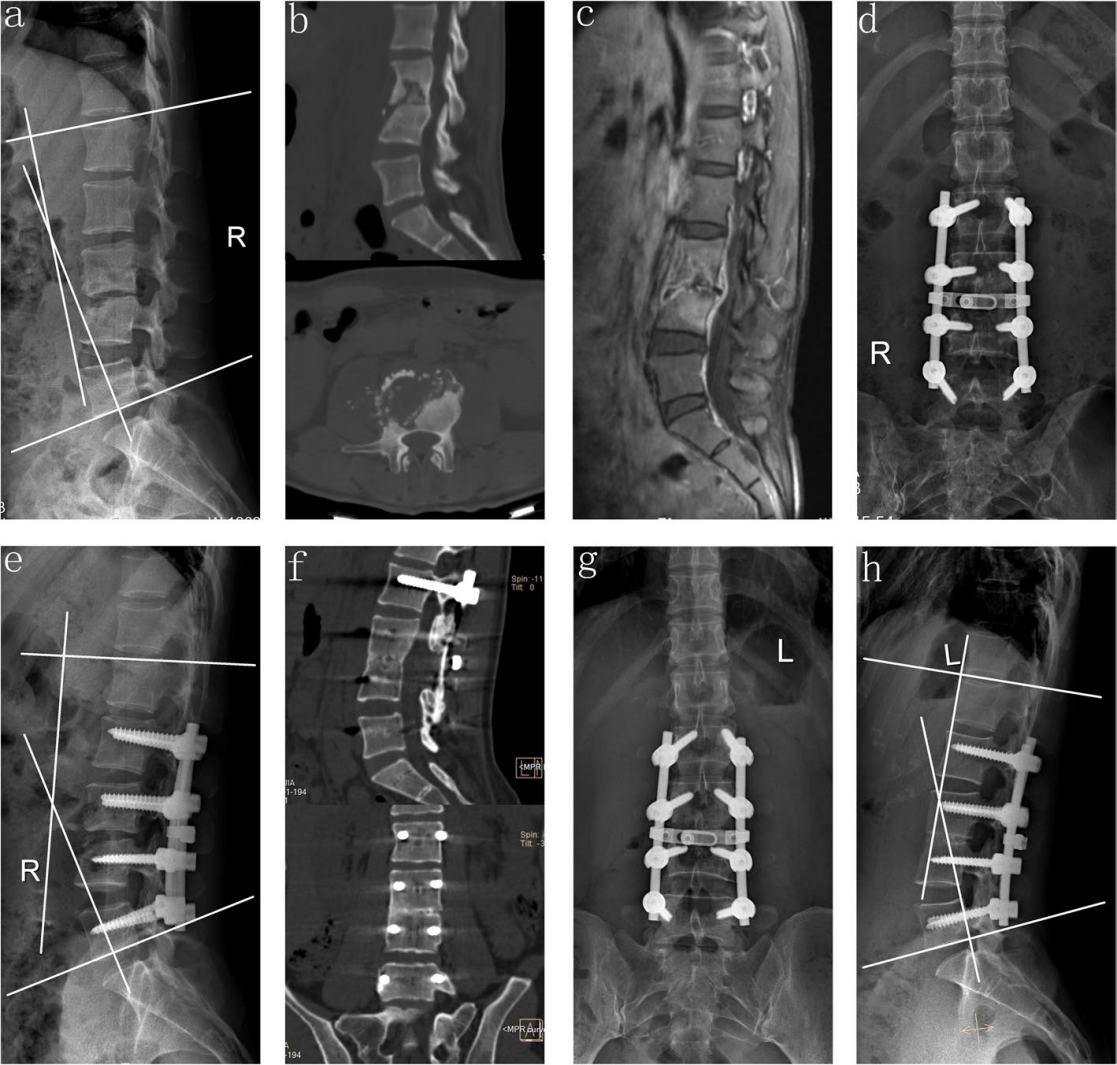
A 32-year-old female who demonstrated lesions received one-stage posterior debridement, autogenous bone interbody fusion, and instrumentation. **(a–c)** Preoperative images showing TB of L3–L4 with the lordosis angle of 11°. (**d–e**) Postoperative X-ray demonstrating correction of the deformity and the lordosis angle was 32°. (**f)** CT showing satisfactory bone fusion at nine months. **(g–h**) X-ray displaying good internal fixation position and solid bone fusion, with correction loss of 3° at the final visit

**Fig. 2:**
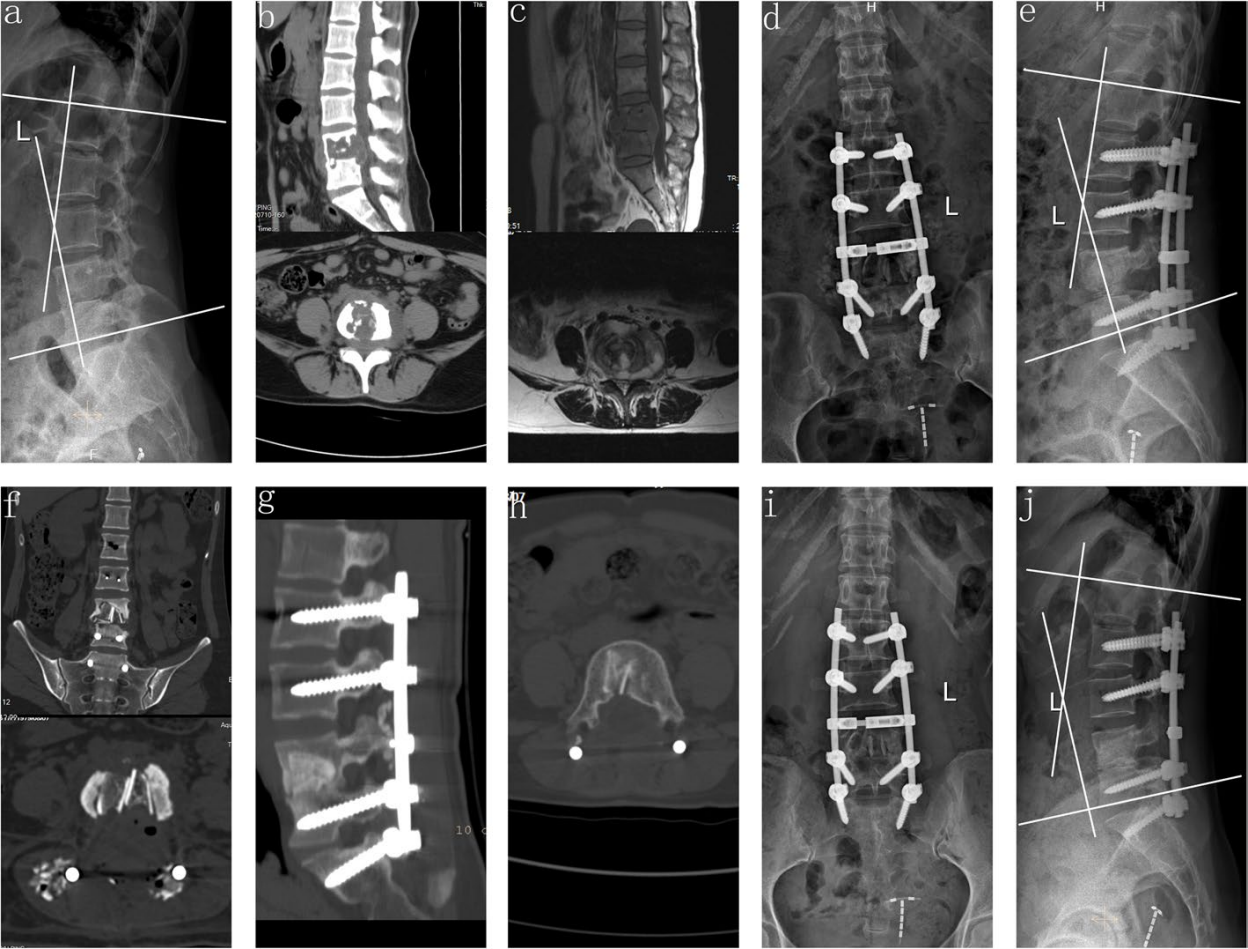
A 33-year-old female demonstrating lesions received one-stage posterior debridement, allogeneic bone interbody fusion, and instrumentation. **(a–c**) Preoperative images showing TB of L4–L5 with deformity (lordosis angle was 19°) and paravertebral abscess formation. (**d–f**) Postoperative X-ray demonstrating correction of the deformity (lordosis angle was 29°) and CT findings of allogeneic bone implanted into the vertebral body. (**g–h**) CT showing satisfactory bone fusion at 24 months. **(i–j)** X-ray displaying good internal fixation position and solid bone fusion, with the lordosis angle of 27° at the 81 months' follow-up

**Fig. 3:**
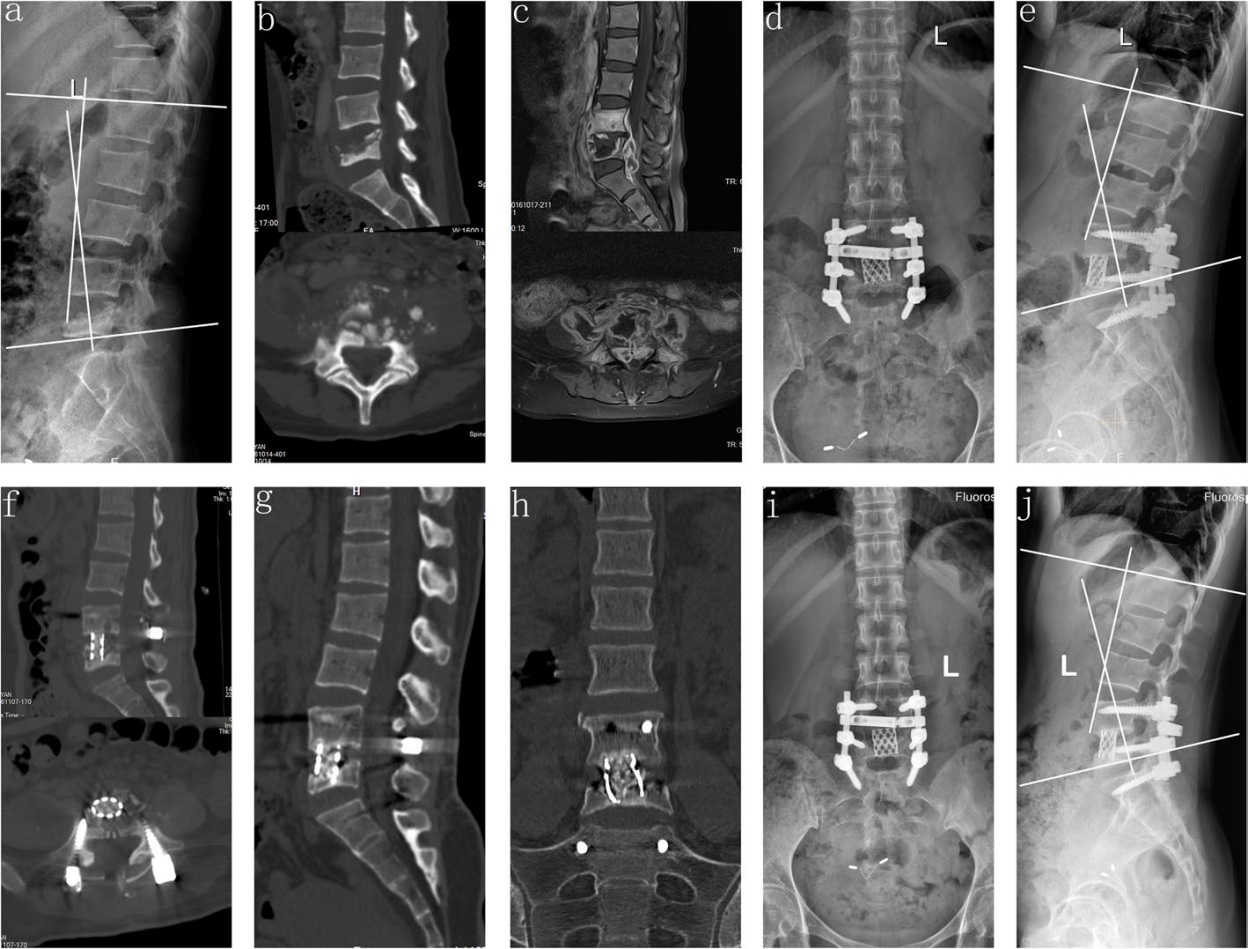
A 42-year-old male who demonstrated lesions received one-stage posterior debridement, one titanium mesh cage bone interbody fusion and instrumentation. **(a–c)**. Preoperative images showing TB of L4–L5 with vertebral body collapse and paravertebral abscess formation; the lordosis angle was 13°. (**d–f**) Postoperative X-ray demonstrating good internal fixation position with the lordosis angle of 28° and CT findings of titanium mesh cage intervertebral bone graft. (**g–h)** CT showing satisfactory bone fusion at nine months. **(i–j**) X-ray displaying solid bone fusion and no collapse or displacement of the cage, with the correction loss of 1°at the final follow-up

The fusion periods of groups A, B, and C, were 9.7 ± 2.4 months, 24.7 ± 4.2 months, and 9.5 ± 2.7 months, respectively, indicating group B's period to be longer than those of the other two groups (Table 2). During the final follow-up, 11 patients in group A had degeneration as per the UCLA grading scale. The same was observed with 13 and 10 patients in groups B and C. There were no statistically significant differences in the rate of ASD among the three groups (Table 3). An imaging examination conducted at the final follow-up indicated that all the grafts were fused (Figs. 1, 2 and 3).

**Table 2:** Postoperative data of patients

	Group A	Group B	Group C	Statistical value	P_A-B_/P_A-C_/P_B-C_
Follow-up period (months)	75.4 ± 11.8	76.5 ± 11.2	76.0 ± 11.5	F = 0.111, *P* = 0.895	-/-/-
Operation period (min)	189.1 ± 27.2	161.8 ± 24.6	163.3 ± 23.3	F = 15.572, *P* = 0.000	< 0.05/ < 0.05/ > 0.05
Intraoperative blood loss (ml)	946.3 ± 185.2	788.9 ± 139.8	777.5 ± 130.6	F = 15.627, *P* = 0.000	< 0.05/ < 0.05/ > 0.05
ESR (mm/h)					
3-month postoperative	11.5 ± 3.0^*^	12.4 ± 3.3^*^	11.9 ± 2.8^*^	F = 0.883, *P* = 0.416	-/-/-
Final follow-up	4.2 ± 1.5^*^	4.7 ± 1.6^*^	4.5 ± 1.8^*^	F = 1.134, *P* = 0.325	-/-/-
CRP (mg/L)					
3-month postoperative	4.5 ± 1.3^*^	4.7 ± 1.2^*^	4.6 ± 1.2^*^	F = 0.259, *P* = 0.772	-/-/-
Final follow-up	1.8 ± 0.7^*^	1.9 ± 0.6^*^	1.9 ± 0.9^*^	F = 0.322, *P* = 0.726	-/-/-
JOA					
Final follow-up	27.1 ± 1.8^*^	27.3 ± 2.0^*^	27.3 ± 1.9^*^	F = 0.158, *P* = 0.854	-/-/-
ODI					
Final follow-up	9.9 ± 1.5^*^	10.0 ± 1.7^*^	10.2 ± 1.7^*^	F = 0.389, *P* = 0.679	-/-/-
VAS					
One day postoperative	8.5 ± 0.9	6.0 ± 1.1	5.9 ± 1.0	F = 85.732, *P* = 0.000	< 0.05/ < 0.05/ > 0.05
Final follow-up	0.9 ± 0.8^*^	0.9 ± 0.7^*^	0.9 ± 0.7^*^	F = 0.074, *P* = 0.929	-/-/-
Lordosis angle (°)					
Postoperative immediately	29.8 ± 4.3^*^	29.6 ± 4.2^*^	30.7 ± 6.8^*^	F = 0.501, *P* = 0.606	-/-/-
Final follow-up	27.0 ± 3.8^*^	26.8 ± 4.6^*^	30.0 ± 6.6^*^	F = 4.877, *P* = 0.009	> 0.05/ < 0.05/ < 0.05
Correction rate (%)	46.5 ± 10.8	46.4 ± 12.5	47.5 ± 7.6	F = 0.138, *P* = 0.872	-/-/-
Correction loss (°)	2.9 ± 1.0	3.1 ± 0.8	0.8 ± 0.7	F = 97.452, *P* = 0.000	> 0.05/ < 0.05/ < 0.05
Fusion period (months)	9.7 ± 2.4	24.7 ± 4.2	9.5 ± 2.7	F = 312.363, *P* = 0.000	< 0.05/ > 0.05/ < 0.05

^*^ Analyzed by paired *t* test, compared with preoperatively, *p* < 0.05

**Table 3:** Preoperative and postoperative UCLA grading scale in three groups

Grade	Group A			Group B			Group C		
	Preoperative	Final follow-up	Degeneration^Δ^	Preoperative	Final follow-up	Degeneration^Δ^	Preoperative	Final follow-up	Degeneration^Δ^
I	34	24	0	37	26	0	32	23	0
II	7	16	10	8	17	11	8	16	9
III	0	1	1	0	2	2	0	1	1
IV	0	0	0	0	0	0	0	0	0

Degeneration^Δ^ indicates one or more grades degeneration compared with preoperative and final follow up. The rate of ASD in groups A, B, and C were 26.8%, 28.9% and 25.0%, respectively. The statistical values were χ^2^ = 0.163 and *P* = 0.922

### Complications

Superficial wound infection was observed to occur in seven cases (3 in group A, 2 in group B, and 2 in group C), which were cured by antibiotics. Three patients in group A complained of postoperative pain in the bone extraction area, which was overcome by administering nonsteroidal anti-inflammatory drugs. Catheter drainage through a minimally invasive incision and regular chemotherapy were procedures through which local abscess recurrence experienced by two cases in group B were treated. Pseudarthrosis was experienced by one case in group B, for which he underwent anterior titanium mesh cage bone grafting.

## Discussion

### Characteristics of lower lumbar spinal TB and its surgical treatment

The lower lumbar spine is at the lowest position of the spine, in which region the body strength is concentrated, specifically at the junction of the active segment and the fixed end. The loading conditions are complex and easily cause force imbalance. The incidence of TB in the lower lumbar spine is insidious, and the symptoms of some patients are atypical. Such individuals often suffer lower back pain, which is easily misdiagnosed as a lumbar disc herniation, lumbar spinal stenosis, osteoporosis, or other degenerative diseases [12]. As the disease develops, *Mycobacterium tuberculosis* erodes the vertebral body to cause dead bones and abscesses, leading to instability or deformity of the spine or invadeing the spinal canal causing neurological symptoms in lower limbs, even cauda equina syndrome. Therefore, solid bone graft fusion and favorable spinal stability are the prerequisites for curing lower lumbar spinal TB and the key to reducing spinal deformity.

The surgical treatment of spinal TB is specifically performed to remove the infection focus, relieve nerve compression, and reconstruct the stability of the spine [13, 14]. Various surgical approaches were adopted to reduce lower lumbar spinal TB [4, 5, 15]. Hodgson and Stock first reported Hong Kong operation (anterior radical surgery) to treat spinal TB in 1960 [16]. However, the majority of TB lesions involve the anterior and middle column; therefore, anterior debridement and bone grafting were recommended by some surgeons [17, 18]. Nevertheless, the long-term effect of the anterior approach showed that the bone graft was susceptible to collapse or absorption, and kyphosis was more severe [19, 20]. To overcome the shortcomings of this method some surgeons adopted anterior and posterior approaches for fixation and fusion, which enhanced the fusion rate of bone graft and the effect of kyphosis correction; however, this surgical procedure significantly increased iatrogenic trauma and hospitalization time, especially the elderly, children, and other physically weak patients [5].

### Advantages of the posterior approach for lower lumbar spinal TB

The posterior-only approach has turned out to be an effective treatment for lower lumbar spinal TB with the advent of posterior spinal instrumentation technology, as reported by several scholars [21–23]. Pedicle screws allow the fixation of the three columns of the spine, effectively restoring the normal physiological curvature of the spine, thus correcting kyphosis, and a better holding force can reduce the risk of loosening and fracture of the internal fixation, and results in a strong biological fixation in a short time post-operation. Intervertebral and intertransverse or interlaminar bone grafting to achieve 360° fusion can ensure the long-term stability of the spine. The rationality of the posterior approach lies in the basic removal of the necrotic tissues and the ossified bone from around the lesion that prevents the entry of anti-TB drugs, thus destroying the positive environment essential for the survival of *Mycobacterium tuberculosis*. The rest of the small number of lesions and abscesses can be absorbed by long-term, standardized anti-TB chemotherapy post-surgery [24]. Pang et al. [25] reported that the posterior approach was comparatively more effective in correcting kyphosis and less traumatic than the anterior approach. The outcomes of the single posterior and anterior–posterior approaches in the treatment of lower lumbar spinal TB were compared by Xu et al. [26], and the posterior procedure was found to be a better one with fewer complications.

### Three bone graft struts for anterior and middle column reconstruction

As per the 3-columns theory of Denis [27], the long-term outcome of the posterior approach depends on the reconstruction of the anterior and middle columns through interbody fusion on the removal of the TB debris. Autogenous bone was considered the gold standard for bone grafting strut due to good biocompatibility and the absence of disease transmission risk [28]. Nevertheless, autogenous bone transplantation cannot satisfy the need for interbody fusion due to complications in the donor site and prolonging the operation time and trauma. Moreover, the number of senile spinal TB patients increased, usually accompanied by osteoporosis and other systemic diseases, such as diabetes and cardiovascular disease, causing low osteoinductive activity and poor osteogenesis of the autogenous bone. The use of allogeneic bone decreases the related complications due to autogenous iliac bone but lacks osteogenic induction ability. Furthermore, the bone block lacks blood circulation, thus hindering the supply of adequate anti-TB drug concentration locally. In this study, operation period and blood loss in group A were greater than those in groups B and C. Two patients of group B suffered local abscess recurrence and were cured by minimally invasive surgery and regular chemotherapy. One patient in group B experienced pseudoarthrosis at the bone graft site and was treated by revision surgery.

Of late, several scholars have evidenced that titanium mesh cage bone graft carries the potential for reliable spine reconstruction, high fusion rate, effective sagittal balance maintenance, and low implant-related complications [29, 30]. Depending on the size and shape of the intervertebral bone defect, one or two shaped titanium mesh cages filled with autogenous bone particles from the healthy lamina and spinous process were implanted. If the bone mass is inadequate, allogeneic bone particles can also be used to fill the middle of the cage. This intervertebral bone grafting technique has its unique advantages. Initially, the cage has sufficient support strength to achieve immediate stability, and is conveniently able to withstand compressive force to prevent it from fracture and displacement. Moreover, implantation of an ideally shaped titanium mesh cage can ensure a relatively large graft volume and the bone contact surface between adjacent vertebral bodies, thus promoting graft fusion in an enhanced way. It also has large load-bearing surfaces, and its mechanical strength is enough to prevent discrete loss of height from a fused motion segment and avoid complications such as subsidence [31]. Eventually, intervertebral implanted titanium mesh can be shaped as per the specific shape of the bone defect, which can retain more healthy bone and prevent complications such as decreased stability of the spine and non-fusion of bone graft as a result of the large bone defect. Certain scholars were concerned that implanting a titanium mesh cage in the lesion area may likely lead to TB recurrence. Nevertheless, it has been demonstrated that TB bacilli have weak adhesion to titanium strut and do not influence the bactericidal effect of anti-TB drugs [32]. Wu et al. [33] reported a structural autograft combined with a titanium mesh cage to treat lumbosacral TB with significant loss of vertebral body, and the results presented that the angle of the lumbosacral was significantly increased from 12.6° preoperatively to 26.4° postoperatively. Zhang et al. [31] compared the efficacy of titanium mesh and autologous bone grafting in treating thoracolumbar spinal TB. They found that the efficacy of titanium mesh was superior to autologous bone grafting. In this study, all the patients belonging to group C successfully attained bone fusion. In contrast the fusion period was significantly less than that in group B and lower correction loss than those of groups A and B.

Since the study encompasses a midterm term follow-up of more than five years post-operation, it is assumed that ASD may occur over time. An earlier biomechanical study indicated that ASD was associated with loss of motor function in the fused segment, a compensatory increase in adjacent segment mobility and mechanical stress, which resulted in augmenting load on the discs and articular processes [34]. Even though interbody fusion is the main cause of ASD, it can also restore the stability of the responsible segment. In this study, the rate of ASD was 26.8% in group A, 28.9% in group B and 25.0% in group C at the final visit. The findings were similar to the incidence of ASD (range 21.3% to 31.9%) after lumbar fusion reported by recent a meta-analysis [35].

This study has the following limitations. First, there could be chances of statistical bias due to the nature of the study, such as a retrospective and single-centre study with relatively small sample size. Therefore, a multicenter study and, a large sample size are warranted.

## Conclusions

Generally, this midterm term follow-up study established that one-stage posterior debridement, interbody fusion, and instrumentation, combined with medical therapy, can effectively treat lower lumbar spinal TB. Moreover, intervertebral titanium mesh cage bone grafts result in better outcomes than autogenous or allogeneic bone grafts.

## Supplementary Information

**Additional file 1**. The datasets used and analyzed in this study.

## Data Availability

All data generated or analyzed during this study are included in this published article (and its supplementary information files).

## Abbreviations

ASD: Adjacent segment degeneration
CRP: C-reactive protein
CT: Computed tomography
MRI: Magnetic resonance imaging
ESR: Erythrocyte sedimentation rate
JOA: Japanese Orthopaedic Association
ODI: Oswestry Disability index
TB: Tuberculosis
UCLA: University of California at Los Angeles
VAS: Visual analogue scale
